# Functional RNA Dynamics Are Progressively Governed by RNA Destabilization during the Adaptation to Chronic Hypoxia

**DOI:** 10.3390/ijms23105824

**Published:** 2022-05-22

**Authors:** Rebekka Bauer, Sofie Patrizia Meyer, Karolina Anna Kloss, Vanesa Maria Guerrero Ruiz, Samira Reuscher, You Zhou, Dominik Christian Fuhrmann, Kathi Zarnack, Tobias Schmid, Bernhard Brüne

**Affiliations:** 1 Faculty of Medicine, Institute of Biochemistry I, Goethe-University Frankfurt, 60590 Frankfurt, Germany; bauer@biochem.uni-frankfurt.de (R.B.); s.meyer@biochem.uni-frankfurt.de (S.P.M.); guerreroruiz@biochem.uni-frankfurt.de (V.M.G.R.); fuhrmann@biochem.uni-frankfurt.de (D.C.F.); b.bruene@biochem.uni-frankfurt.de (B.B.); 2 Faculty of Biological Sciences, Buchmann Institute for Molecular Life Sciences (BMLS), Goethe-University Frankfurt, 60438 Frankfurt, Germany; karo.kloss@googlemail.com (K.A.K.); samira.reuscher@gmail.com (S.R.); you.zhou@bmls.de (Y.Z.); 3 German Cancer Consortium (DKTK), Partner Site Frankfurt, 60590 Frankfurt, Germany; 4 Frankfurt Cancer Institute, Goethe-University Frankfurt, 60596 Frankfurt, Germany; 5 Fraunhofer Institute for Translational Medicine and Pharmacology, 60596 Frankfurt, Germany

**Keywords:** hypoxia, monocytes, de novo transcription, RNA stability, SLAM-seq, GRAND-SLAM

## Abstract

Previous studies towards reduced oxygen availability have mostly focused on changes in total mRNA expression, neglecting underlying transcriptional and post-transcriptional events. Therefore, we generated a comprehensive overview of hypoxia-induced changes in total mRNA expression, global de novo transcription, and mRNA stability in monocytic THP-1 cells. Since hypoxic episodes often persist for prolonged periods, we further compared the adaptation to acute and chronic hypoxia. While total mRNA changes correlated well with enhanced transcription during short-term hypoxia, mRNA destabilization gained importance under chronic conditions. Reduced mRNA stability not only added to a compensatory attenuation of immune responses, but also, most notably, to the reduction in nuclear-encoded mRNAs associated with various mitochondrial functions. These changes may prevent the futile production of new mitochondria under conditions where mitochondria cannot exert their full metabolic function and are indeed actively removed by mitophagy. The post-transcriptional mode of regulation might further allow for the rapid recovery of mitochondrial capacities upon reoxygenation. Our results provide a comprehensive resource of functional mRNA expression dynamics and underlying transcriptional and post-transcriptional regulatory principles during the adaptation to hypoxia. Furthermore, we uncover that RNA stability regulation controls mitochondrial functions in the context of hypoxia.

## 1. Introduction

Hypoxia is a common environmental factor both in physiological and pathophysiological contexts. Specifically, while high altitudes as well as certain cellular niches, such as the bone marrow, are inherently characterized by low oxygen tensions, hypoxic conditions pathologically occur, e.g., upon vascular thrombosis, within rapidly growing tumors, as well as in diseases associated with severe inflammatory conditions [1,2]. Since ambient oxygen availability is critical for numerous cellular processes, such as energy production by mitochondrial oxidative phosphorylation, it is not surprising that adaptive processes to hypoxia have been in the limelight of research for many decades [3]. Specifically, hypoxia induces a rapid increase in glucose transporters and glycolytic enzymes to ensure sufficient energy supply and, furthermore, even represses mitochondrial function [4,5]. Under prolonged hypoxic conditions this metabolic rewiring appears to be further stabilized by autophagy-dependent removal of the mitochondria [6,7,8].

Nevertheless, despite the fact that disease conditions commonly reflect conditions of chronic hypoxia, efforts to molecularly characterize hypoxia responses have so far largely focused on acute hypoxic conditions, identifying the oxygen-sensitive transcription factors hypoxia-inducible factor (HIF) 1 and 2 as key regulators of adaptive processes [9,10,11]. Interestingly though, during extended hypoxia, both HIFs are downregulated again [12,13], which in combination with a new steady state expression of classical hypoxia response genes, was recently put forward as an indicator for chronic hypoxia [14,15]. Importantly, this definition of chronic hypoxia encompasses a cellular response state rather than an exact duration of hypoxic conditions, and was detected in THP-1 monocytes as well as in various multiple myeloma after 72 h of hypoxia [14,15]. Notably, in line with the decreasing relevance of HIF-mediated adaptations during prolonged hypoxia, post-transcriptional regulatory mechanisms, including mRNA stability regulation, translational changes, and alternative splicing, have been shown to contribute to hypoxic responses as well [12,16,17,18,19].

## 2. Results

As the contribution of different regulatory layers on gene expression changes throughout the course of hypoxia remains largely elusive, we aimed at characterizing altered mRNA dynamics during acute and chronic oxygen deprivation. To this end, we exposed THP-1 monocytes to acute (AH, 8 h, 1% oxygen) and chronic hypoxia (CH, 72 h, 1% oxygen) [14] and employed SLAM-seq (thiol(SH)-linked alkylation for the metabolic sequencing of RNA) [20]. Specifically, we added 4-thiouridine (4sU; 300 µM) during the last hour of the respective incubations to label newly synthesized mRNA and determined differential gene expression (DGE) changes and differentially de novo synthesized (DDNS) mRNAs by RNA sequencing based on changes in total read counts and reads harboring T-to-C transitions due to 4sU incorporation, respectively.

### 2.1. Hypoxia Induces Dynamic Changes in mRNA Expression

**Figure 1:**
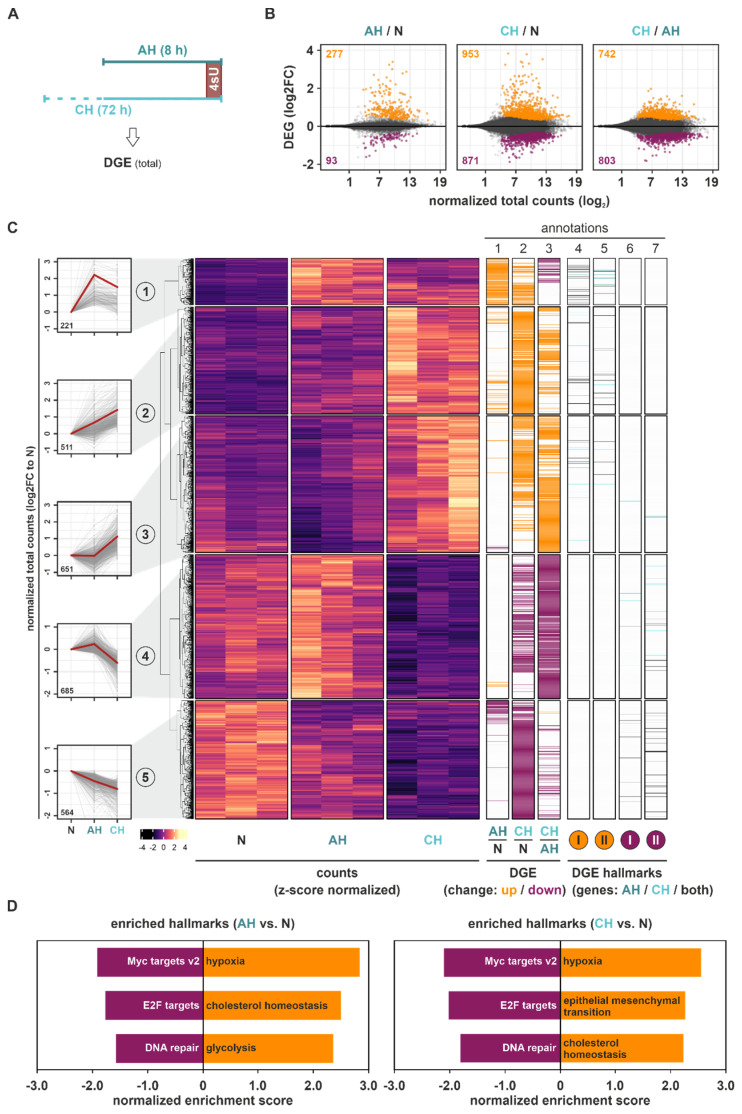
Differential gene expression (DGE) changes under hypoxia. (**A**) THP-1 cells were incubated under hypoxia (1% oxygen) for 8 h (acute hypoxia, AH) or 72 h (chronic hypoxia, CH), or under normoxia, supplemented with 300 µM 4-thiouridine (4sU) for the last hour. DGE changes were determined based on changes in total read counts. (**B**) MA plots representing DGE changes (log2FC) between AH and N (*left*), CH and N (*middle*), or CH and AH (*right*). Significant DGE targets (padj < 0.05) are indicated in orange (up) or purple (down). (**C**) Heatmap representing *z*-score-normalized counts of all DGE targets significantly altered in at least one of the comparisons. Five groups representing different DGE dynamics were identified by *k*-means clustering analysis (*left panels*). Annotation columns 1–3 depict DGE changes (up: orange; down: purple) for the comparison of AH and N, CH and N, and CH and AH. Annotation columns 4–7 depict targets contributing to the GSEA-enriched hallmarks either upregulated (I: hypoxia; II: cholesterol homeostasis (*orange*)) or downregulated (I: Myc targets v2; II: E2F targets (*purple*)) in DGE of either AH vs. N, CH vs. N, or both. (**D**) Top three enriched up- or downregulated hallmarks identified by GSEA of total mRNA expression under AH or CH relative to N.

Initially, we focused on global gene expression changes in THP-1 monocytes from acute to extended hypoxic exposures (Figure 1A). Overall, more pronounced DGE changes compared to normoxia (N) (Benjamini–Hochberg-corrected *p*-value (padj) < 0.05) occurred under CH than under AH (DGE(CH/N): 1824; DGE(AH/N): 370) (Figure 1B). The observation that a large proportion of the additional genes regulated under CH significantly changed between CH and AH, supports the notion that a major part of the adaptive processes to hypoxia is induced under extended hypoxic incubations only. To obtain further insights into the expression dynamics under hypoxia, we used *k*-means clustering analysis of all DGE targets significantly regulated between AH, CH, and/or N, identifying five clusters of targets following different expression patterns in the course of hypoxia (Figure 1C (*left panels*); Figure S1): A small group of DGE targets (8% of all DGE; cluster 1) rapidly increased already under AH, remaining stable or decreasing under CH (Figure 1C (*annotation columns 1–3*)). Two larger groups of genes steadily increased (cluster 2) or decreased (cluster 5) in expression across the course of hypoxia, representing 19% and 21% of all DGE changes, respectively. In line with the above described observation that CH induced more substantial DGE changes, the two largest clusters were characterized by a predominant or exclusive up- (cluster 3; 25%) or downregulation (cluster 4; 26%) under CH. Notably, while the mean absolute read counts of genes appeared to be similar across all clusters, targets in cluster 4 (predominantly downregulated under CH) appeared to be expressed at higher basal (N) levels (Figure S1; Table S1). 

To assess potential functional implications of the altered mRNA expression profiles in the course of hypoxia, gene set enrichment analyses (GSEA) for specific well-defined biological states or processes (hallmarks) were carried out for all mRNA expression changes occurring under AH and CH relative to N. Despite the marked difference in DGE changes between AH and CH, the same functional implications were enriched in both conditions (Table S2), i.e., hypoxia and cholesterol homeostasis emerged among the top enriched upregulated hallmarks, while Myc targets, E2F targets, and DNA repair were among the top enriched downregulated hallmarks (Figure 1D and Figure S2).

In line with this, many of the DGE targets constituting the enriched hallmarks hypoxia and cholesterol homeostasis were upregulated, while Myc targets and E2F targets were downregulated in both AH and CH. A few DGE candidates associated with these functional hallmarks appeared to be altered exclusively under CH, mostly in clusters 3 and 4 (Figure 1C (*annotation columns 4–7*)).

Taken together, total mRNA expression changes during the course of hypoxia not only bore signs of enhanced classical hypoxia responses, but also were enriched for upregulated cholesterol metabolism and reduced Myc and E2F target expression. This suggests altered metabolic processes as well as reduced proliferation and cell cycle progression. The finding that chronic conditions generally induced more pronounced DGE changes than AH, and that the proportion of downregulated targets substantially increased under CH, implies that the regulatory principles underlying dynamic mRNA expression changes differ considerably between short- and long-term hypoxia.

### 2.2. Hypoxia Enhances Transcriptional Responses

**Figure 2:**
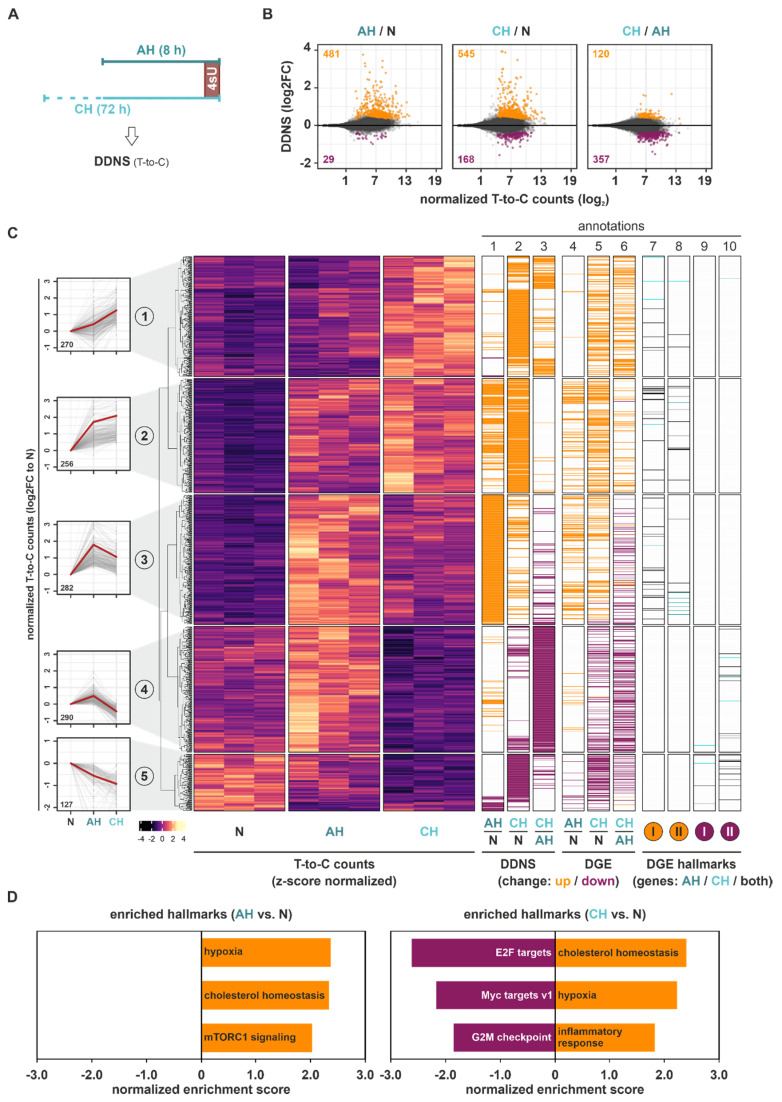
Differential de novo synthesis (DDNS) changes under hypoxia. (**A**) THP-1 cells were incubated under hypoxia (1% oxygen) for 8 h (acute hypoxia, AH) or 72 h (chronic hypoxia, CH), or under normoxia, supplemented with 300 µM 4-thiouridine (4sU) for the last hour. DDNS changes were determined based on 4sU alkylation-dependent changes in T-to-C conversions. (**B**) MA plots representing DDNS changes (log2FC) between AH and N (*left*), CH and N (*middle*), or CH and AH (*right*). Significant DDNS targets (padj < 0.1) are indicated in orange (up) or purple (down). (**C**) Heatmap reflecting *z*-score-normalized T-to-C read counts of all DDNS targets significantly altered in at least one of the comparisons. Five groups representing different DDNS dynamics were identified by *k*-means clustering analysis (*left panels*). Annotation columns 1–3 contain DDNS changes (up: orange; down: purple) for the comparison of AH and N, CH and N, and CH and AH. Annotation columns 4–6 encompass DGE changes (up: orange; down: purple) for the comparison of AH and N, CH and N, and CH and AH. Annotation columns 7–10 reflect targets from the GSEA-enriched hallmarks either upregulated (I: hypoxia; II: cholesterol homeostasis) or downregulated (I: Myc targets v2; II: E2F targets) in DGE of either AH, CH, or both (Figure 1D). (**D**) Top three enriched up- or downregulated hallmarks identified by GSEA of T-to-C count changes under AH or CH relative to N.

In order to obtain further insights into the regulatory mechanisms governing the observed changes in total RNA dynamics, we next analyzed newly transcribed mRNAs (DDNS) by assessing T-to-C conversions resulting from chemical modification of incorporated 4sU in newly synthesized mRNAs (Figure 2A and Figure S3A). The vast majority of AH- or CH-induced DDNS changes (padj < 0.1) reflected the enhanced transcription of targets, i.e., 94% and 76% of DDNS targets were upregulated under AH and CH, respectively (Figure 2B). Further comparison of the DDNS changes between CH and AH revealed only a few additional changes, pointing to the attenuation of hypoxia-induced transcription under chronic conditions. Alike to the DGE changes, *k*-means clustering of all DDNS targets regulated between AH, CH, and/or N identified five groups of targets representing different DDNS dynamics in the course of hypoxia (Figure 2C (*left panels*); Figure S4 (*upper panels*)). Three similarly-sized clusters, representing 66% of significant DDNS changes, corresponded to targets with enhanced transcription during hypoxia, either steadily increasing across the course of hypoxia (cluster 1) or alternatively increasing under AH, and thereafter remaining stable (cluster 2) or decreasing again (cluster 3). Clusters 4 (24%) and 5 (10%) depicted DDNS that were transcriptionally downregulated either exclusively under CH (cluster 4) or cumulatively throughout the hypoxic exposure (cluster 5) (Figure 2C (*annotation columns 1–3*); Table S3). Notably, transcriptional changes (Figure 2C (*annotation columns 1–3*)) in the identified DDNS targets appeared to closely resemble their changes in DGE level (Figure 2C (*annotation columns 4–6*)). Moreover, the increase in normalized T-to-C read counts under basal (N) conditions observed from DDNS clusters 1 to 4, which decreased again in cluster 5, closely followed the corresponding changes in total mRNA expression (Figure S4).

In line with the concurrent changes in DDNS and DGE, GSEA of the DDNS changes also identified hypoxia and cholesterol homeostasis as the top enriched upregulated hallmarks under AH and CH (Figure 2D and Figure S5; Table S4). Due to the low number of downregulated DDNS targets, no enriched downregulated hallmarks were identified in DDNS under AH, yet E2F and Myc targets were again enriched under CH.

Thus, the analysis of changes in de novo mRNA synthesis suggested marked transcriptional adaptations under AH with only minor additional changes under CH. The enhanced transcription of hypoxia- and cholesterol metabolism-associated targets corroborated the changes in DGE level, while downregulated DDNS correlated with the DGE changes under CH related to cell cycle-associated hallmarks.

### 2.3. Hypoxia Reduces RNA Stability

**Figure 3:**
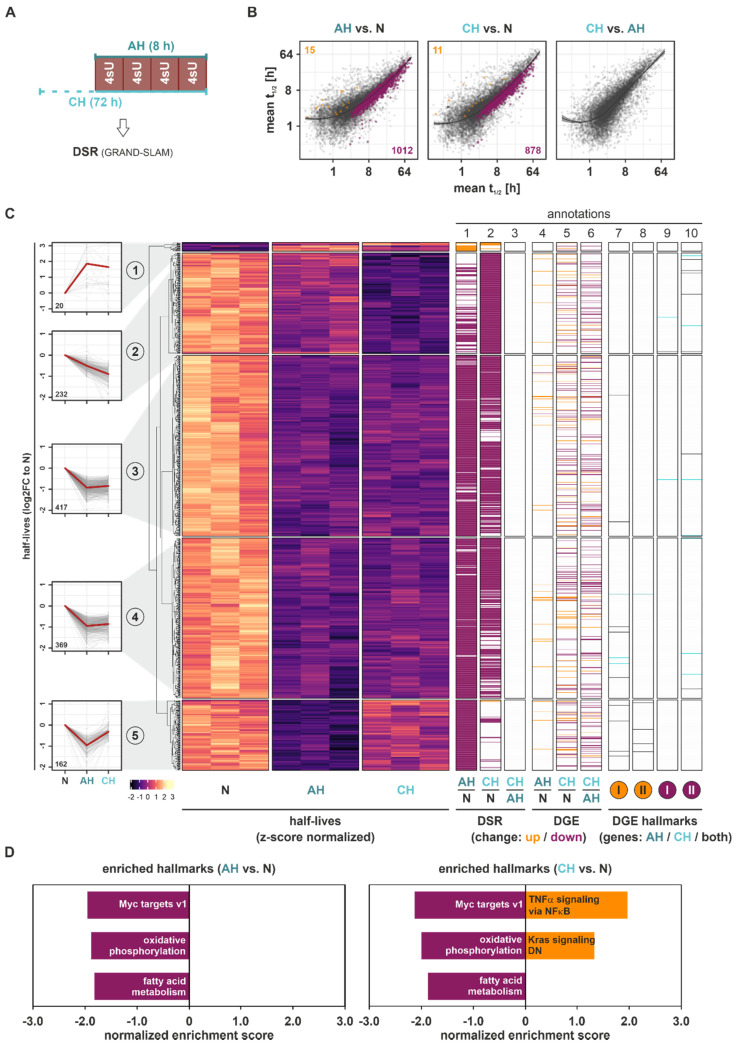
Differential stability regulation (DSR) changes under hypoxia. (**A**) THP-1 cells were incubated under hypoxia (1% oxygen) for 8 h (acute hypoxia, AH) or 72 h (chronic hypoxia, CH), or under normoxia, supplemented with 4 × 30 µM 4-thiouridine (4sU) during the last 8 h. DSR changes were determined based on 4sU alkylation-dependent changes in T-to-C conversions using GRAND-SLAM. (**B**) Scatter plots comparing mean mRNA half-lives at AH vs. N (*left*), CH vs. N (*middle*), and CH vs. AH (*right*) highlighting significantly increased (orange) or decreased (purple) half-lives between the conditions (padj < 0.1, local linear regression + 95% confidence intervals). (**C**) Heatmap reflecting *z*-score-normalized half-lives of all DSR targets significantly altered in at least one of the comparisons. Five groups representing different DSR dynamics were identified by *k*-means clustering analysis (*left panels*). Annotation columns 1–3 contain DSR changes (up: orange; down: purple) for the comparison of AH and N, CH and N, and CH and AH. Annotation columns 4–6 encompass DGE changes (up: orange; down: purple) for the comparison of AH and N, CH and N, and CH and AH. Annotation columns 7–10 reflect targets contributing to the GSEA-enriched hallmarks either upregulated (I: hypoxia; II: cholesterol homeostasis) or downregulated (I: Myc targets v2; II: E2F targets) in DGE of either AH, CH, or both (Figure 1D). (**D**) Top three enriched up- or downregulated hallmarks identified by GSEA of mRNA half-life changes under AH or CH relative to N.

As transcriptional changes (DDNS) appeared to explain only parts of the total gene expression changes (DGE) under hypoxia, and since post-transcriptional modes of regulation, including mRNA stability regulation, are known to commonly contribute to gene expression changes in response to extended stimulations [21,22], we next determined differentially stability regulated (DSR) mRNAs under hypoxic conditions. Therefore, we labeled THP-1 cells for 8 h with 4sU, i.e., four pulses of 30 µM 4sU every 2 h during the last 8 h of the experiment to ensure sufficient labeling (Figure 3A). Indeed, 4 × 30 µM 4sU administered over a time course of 8 h strongly increased T-to-C conversion rates compared to a single dose of 300 µM, while it did not affect the viability of THP-1 cells (Figure S3B,C). mRNA half-lives were determined based on T-to-C conversions using the GRAND-SLAM pipeline [23]. Interestingly, not only were global mRNA half-lives significantly reduced under AH (median half-life = 3.30 h) and CH (3.16 h) as compared to N (3.71 h) (Figure S6A), but the vast majority (99%) of DSR changes in response to both AH and CH compared to N (padj < 0.1) corresponded to reduced mRNA stability, with only a few stabilized targets (Figure 3B). The stratification of all DSR targets comparing AH, CH, and/or N by *k*-means clustering again identified five groups representing different half-life dynamics across the course of hypoxia (Figure 3C (*left panels*); Figure S7 (*upper panels*)). Only 2% of DSR targets displayed enhanced half-lives in response to hypoxia, which appeared to transiently increase under AH only (cluster 1). All other groups were characterized by reduced mRNA half-lives already under AH. Specifically, cluster 2 represented targets with a steady reduction in target half-life from AH to CH. Despite their separation by clustering analysis, clusters 3 and 4, representing 65% of all DSR targets, showed markedly reduced mRNA half-lives under AH, which remained low under CH. Cluster 5 contained DSR targets with a strong, yet transient reduction in half-lives during the course of hypoxia (Figure 3C (*annotation columns 1–3*); Table S5). In contrast to the distribution in DDNS, DSR changes (Figure 3C (*annotation columns 1–3*)) poorly correlated with changes in DGE level (Figure 3C (*annotation columns 4–6*)).

Notably, the few targets showing an increase in half-lives under hypoxia (cluster 1) shared rather short basal (N) half-lives (median half-life = 1.41 h), whereas the targets in cluster 5 (transient decrease in half-lives) had the longest basal (N) half-lives (16.89 h), and clusters 2–4 ranked at 7.49 h, 12.31 h, and 11.15 h, respectively (Figure S7).

In line with the observation that downregulated DSR targets vastly outnumbered upregulated ones, the GSEA of mRNA half-lives identified predominantly downregulated hallmarks (Figure 3D and Figure S8; Table S6). Specifically, Myc targets, oxidative phosphorylation, and fatty acid metabolism emerged as the top enriched downregulated hallmarks under both AH and CH, and only TNFα signaling via NFκB and downregulated Kras signaling appeared to be enriched in upregulated DSR under CH.

In summary, our data on the differential stability regulation of mRNAs in the course of hypoxia indicated that most DSR targets are destabilized under hypoxia and that the major regulated groups show only slight changes between acute and chronic conditions. Enriched hallmarks within the downregulated DSR targets pointed to altered Myc activation and adaptation of metabolic processes (oxidative phosphorylation, fatty acid metabolism) under both AH and CH.

### 2.4. Functional DDNS and DSR Determine Specific Changes in DGE Dynamics in Hypoxia

**Figure 4:**
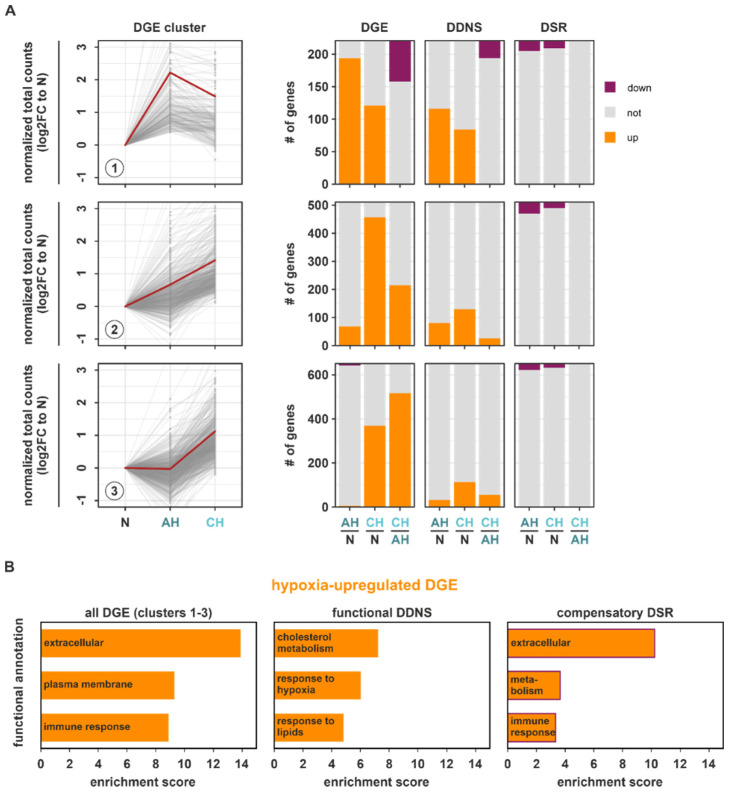
Impact of DDNS and DSR on upregulated DGE targets during hypoxia. (**A**) Changes in the upregulated DGE target clusters (1–3) on the level of DGE, DDNS, and DSR comparing AH and N, CH and N, and CH and AH are depicted as stacked bar graphs. (**B**) Top enriched functional annotations in all upregulated DGE within clusters 1–3 (*left panel*), in functional DDNS, i.e., genes which are upregulated on DGE and DDNS level (*middle panel*), and in compensatory DSR, i.e., genes which are upregulated on DGE and downregulated on DSR level (*right panel*) as determined by DAVID.

Having established differential regulatory patterns for transcriptional and stability regulation under hypoxia and the divergent correlation with the prime enriched DGE hallmarks, we next assessed which of the identified DDNS and DSR changes might influence total gene expression (DGE). First, we focused on DGE targets upregulated in the course of hypoxia (DGE clusters 1–3) and determined how DGE targets within these clusters behaved on DDNS and DSR levels (Figure 4A; Table S7). Herein, DDNS changes closely resembled the DGE changes, whereas DSR changes appeared to rather counteract the DGE changes, i.e., upregulated mRNAs at DDNS and DGE level were destabilized at the DSR level at the same time. Furthermore, DDNS overlapped better with DGE targets induced already under AH (clusters 1 and 2), suggesting the early induction of transcriptional responses. To assess which adaptive cellular processes induced under hypoxia might be regulated at a transcriptional or a post-transcriptional level, we determined enriched functional annotations in upregulated DGE targets concomitantly upregulated in DDNS (functional DDNS) or in upregulated DGE targets downregulated in DSR (compensatory DSR). In line with the observation that DGE changes representing the enriched hallmarks hypoxia and cholesterol homeostasis (Table S2) were also found in the upregulated DDNS clusters 1–3 (Figure 2C (*annotation columns 7–8*)), functional DDNS were enriched for cholesterol metabolism and hypoxia response (Figure 4B (*middle panel*); Table S8) as identified for all DGE (Figure 1D) and DDNS changes (Figure 2D). Instead, upregulated DGE (clusters 1–3) showed an enrichment of rather general annotations referring to altered membrane composition and communication with the microenvironment (extracellular, immune response) (Figure 4B (*left panel*); Table S8). Interestingly, functional annotations enriched within the compensatory DSR indeed appeared to counteract the DGE-associated functions’ extracellular and immune response (Figure 4B (*right panel*); Table S8). Collectively, these observations suggested that adaptive processes induced by hypoxia are facilitated by transcriptional programs, while mRNA stability changes rather appear to counteract enhanced functional programs.

**Figure 5:**
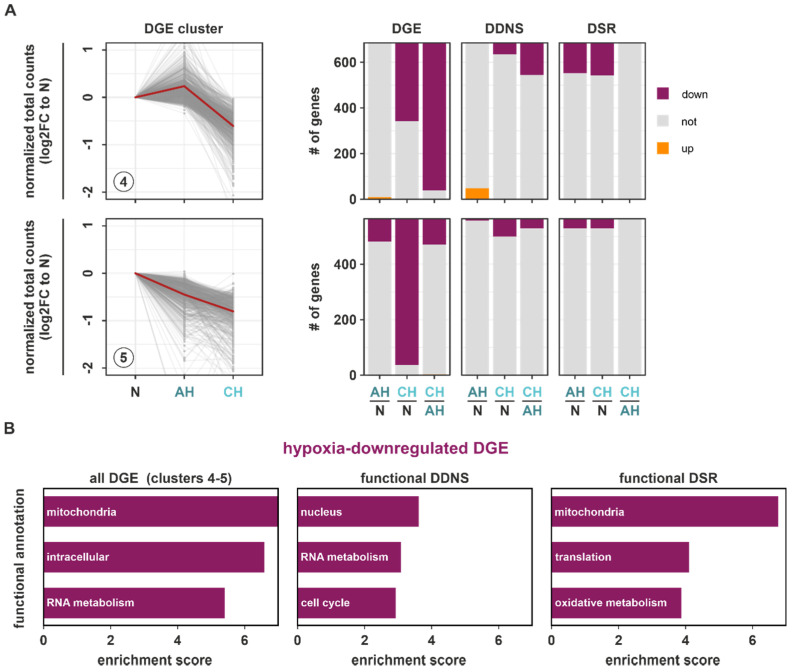
Impact of DSR and DDNS on downregulated DGE targets during hypoxia. (**A**) Changes in the downregulated DGE target clusters (4–5) on the level of DGE, DDNS, and DSR comparing AH and N, CH and N, and CH and AH are depicted as stacked bar graphs. (**B**) Top enriched functional annotations in all downregulated DGE within clusters 4–5 (*left panel*), in functional DDNS, i.e., genes which are downregulated on DGE and DDNS level and unaltered in DSR (*middle panel*), and in functional DSR, i.e., genes which downregulated on DGE and on DSR level and unaltered in DDNS (*right panel*) as determined by DAVID.

We next asked how downregulated DGE targets (DGE clusters 4 and 5) changed at the DDNS and DSR levels (Figure 5A; Table S7). Generally, downregulated DGE targets overlapped to a much smaller extent with both DDNS and DSR changes. Furthermore, downregulation of DGE targets and concomitant DDNS changes mostly occurred under chronic conditions. In contrast, DSR changes corresponding to downregulated DGE targets were observed similarly under AH and CH. As most DDNS and DSR changes corresponded to downregulated DGE in clusters 4 and 5, only targets decreasing exclusively on DDNS (functional DDNS) or DSR level (functional DSR) in parallel to downregulated DGE were used for the subsequent functional enrichment analyses to allow for an unambiguous assignment to either transcriptional or post-transcriptional regulatory processes. Functional DDNS were enriched in the nucleus, RNA metabolism, and cell cycle annotations (Figure 5B (*middle panel*); Table S8), which corroborated that DGE targets constituting the enriched downregulated DGE hallmarks associated with the cell cycle and transcription (Table S2) were also found in the downregulated DDNS clusters 1–3 (Figure 2C (*annotation columns 9–10*)). Thus, transcriptional changes appeared to correlate well with global gene expression changes. In contrast, the hallmarks enriched in global DGE changes were only poorly reflected on DSR levels (Figure 3C (*annotation columns 7–10*)). Interestingly though, functional DSR targets showed a massive enrichment in mitochondrial functions, and to a lesser extent in translation and oxidative metabolism (Figure 5B (*right panel*); Table S8), corroborating the functional annotation with the highest enrichment in all downregulated DGE targets within clusters 4 and 5 (Figure 5B (*left panel*); Table S8). STRING analysis further supported the tight connection of mitochondria and metabolic changes within the hypoxia-downregulated functional DSR targets (Figure S9; Table S9).

Taken together, general adaptations during the course of hypoxia appear to be determined largely at a transcriptional level. Yet, our data further suggest that post-transcriptional regulatory principles, i.e., mRNA destabilization, also might play an important role. The later may act, e.g., by limiting the extent of hypoxia-induced immune responses, but also by implementing a reduction in nuclear-encoded, mitochondria-associated mRNAs (see below), thereby contributing to the establishing of specific adaptive processes to (extended) hypoxic conditions.

### 2.5. Nuclear-Encoded Mitochondrial mRNAs Are Destabilized in the Course of Hypoxia

A marked reduction in mitochondria in chronic hypoxia in THP-1 cells was previously shown to be mediated by the autophagic removal of mitochondria, i.e., mitophagy. Moreover, the expression of numerous mitochondrial proteins involved in mitochondrial membranes and respiratory chain complexes was also strongly reduced, though not exclusively associated to autophagy [14]. In line with this, we observed reduced mitochondrial mass and oxygen consumption rates, representing mitochondrial function, in THP-1 cells under CH (Figure S10). We therefore asked if mRNAs encoding mitochondrial proteins are indeed destabilized during hypoxia. This may prevent the futile production of novel mitochondria under conditions where excess mitochondrial activity would not only be limited due to reduced oxygen availability, but instead might be deleterious to cells due to the uncontrolled production of reactive oxygen species [24].

**Figure 6:**
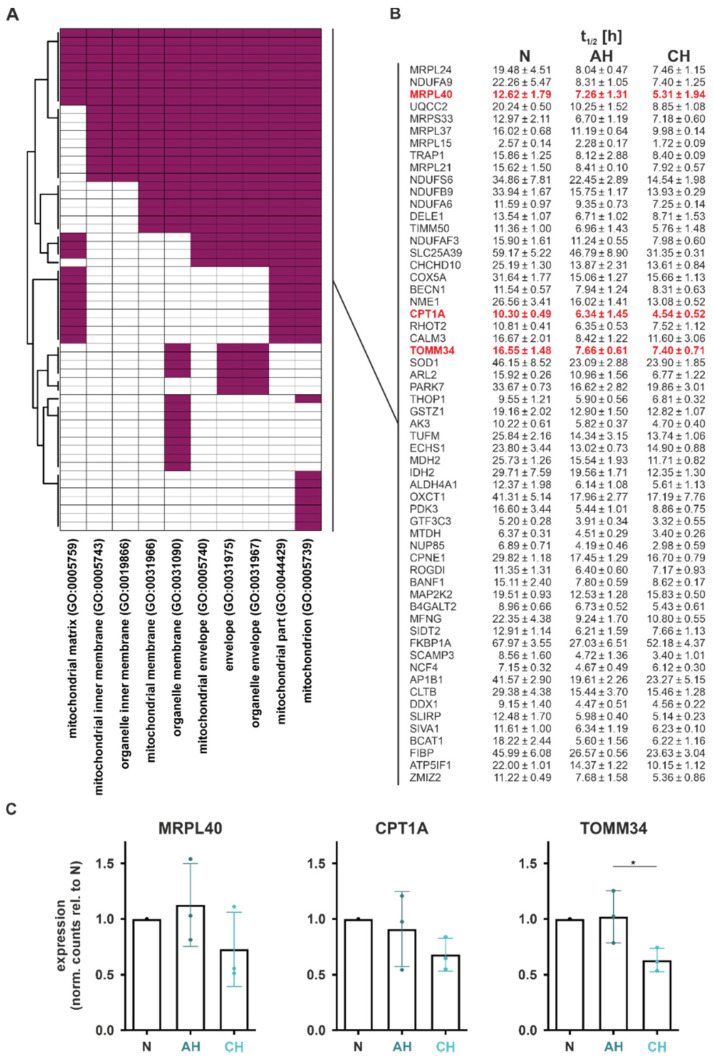
Validation of functional DSR during hypoxia associated with mitochondria. (**A**) GO terms corresponding to mitochondrial functions (Figure 5) and the contributing hypoxia-regulated functional DSR. (**B**) Half-lives of hypoxia-regulated functional DSR under N, AH, or CH as determined by GRAND-SLAM. (**C**) DGE changes in selected functional DSR. Residuals were tested for normality using Shapiro–Wilk test, and one-way ANOVA with Tukey’s multiple comparison test was performed (* *p* < 0.05).

In total, 59 functional DSR targets within 10 GO terms comprised the enriched functional annotation mitochondria, termed functional mito-DSR targets (Figure 6A). Interestingly, the median half-life of these functional mito-DSR targets was reduced from 16.02 h under normoxia to 8.31 h under both AH and CH (Figure 6B).

In order to validate the half-life changes in mitochondrial targets, we selected the mRNAs encoding MRPL40 (mitochondrial ribosomal protein L40), CPT1A (carnitine palmitoyltransferase 1A), and TOMM34 (translocase of outer mitochondrial membrane 34) for further analyses, based on their association with different mitochondrial functions. While reduced half-lives were already observed under AH for all candidates (t_1/2_ (AH relative to N): *MRLP40*: 58%; *CPT1A*: 62%; *TOMM34*: 46%), functional consequences, i.e., the decrease in total mRNA levels, were appreciable only under CH (Figure 6C).

**Figure 7:**
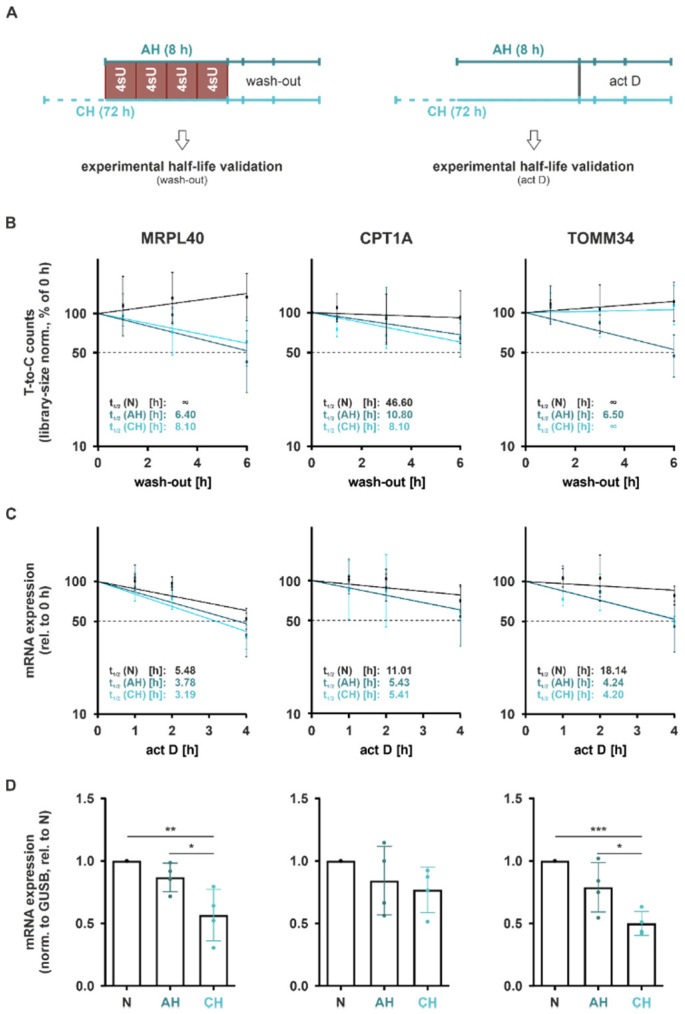
Validation of altered functional mRNA stability under hypoxia. (**A**) THP-1 cells were incubated under hypoxia (1% oxygen) for 8 h (acute hypoxia, AH) or 72 h (chronic hypoxia, CH), or under normoxia. For experimental validation of half-life changes, cells were supplemented with 4 × 30 µM 4-thiouridine (4sU) during the last 8 h, followed by a wash-out with excess uridine of up to 6 h and RNA sequencing (*left*). Alternatively, transcription was blocked by the addition of actinomycin D (2.5 µg/mL; act D) at the end of the incubations, and mRNA expression was followed for up to 4 h by RT-qPCR analyses (*right*). (**B**) mRNA stability of selected functional DSR was determined by wash-out analyses. Library-size corrected T-to-C counts were normalized to 0 h wash-out. (**C**) mRNA stability of selected functional DSR was further analyzed by following mRNA levels after transcriptional blockade. mRNA expression was assessed by RT-qPCR. Half-lives (t_1/2_) were determined by linear regression (**B**,**C**). (**D**) Total mRNA expression of selected functional DSR after AH or CH as compared to N was assessed by RT-qPCR. Residuals were tested for normality using Shapiro–Wilk test, and one-way ANOVA with Tukey’s multiple comparison test was performed (*n* ≥ 3; * *p* < 0.05, ** *p* < 0.01, *** *p* < 0.001).

Considering that these half-lives were determined using a wash-in strategy, i.e., estimating half-lives based on the contribution of mRNA synthesis and decay (GRAND-SLAM), we further aimed to validate the half-lives in an orthogonal wash-out experiment. In this setting, we followed the decay of mRNAs, i.e., the remaining T-to-C conversions for up to 6 h after removal of 4sU and the addition of excess uridine (Figure 7A (*left*)). Notably, half-lives determined using the wash-out approach appeared to be substantially longer than those predicted by GRAND-SLAM (Figure 7B). Moreover, while the so-determined half-lives of *MRPL40* and *CPT1A* reflected a destabilization under AH and CH, the half-life reduction in *TOMM34* was only observed under AH (Figure 7B (*right panel*)).

To obtain independent experimental evidence on the half-lives, we next blocked de novo mRNA synthesis by adding the transcription inhibitor actinomycin D (2.5 µg/mL; act D) at the end of incubations under N, AH, or CH and followed mRNA levels of the selected candidates for up to 4 h (Figure 7A (*right*)). As seen before, half-lives were already substantially reduced under AH, and remained low or slightly increased again under CH (Figure 7C). This resulted in a marked downregulation in total mRNA expression of all candidates under CH and to a minor degree already under AH (Figure 7D). Interestingly, half-lives determined using this approach resembled the GRAND-SLAM-calculated half-lives much closer than the wash-out data.

In summary, we provide evidence that nuclear-encoded mRNAs of mitochondrial proteins are indeed downregulated under chronic hypoxia by a reduction in their mRNA stability. These changes appear to be initiated already under acute hypoxia, suggesting that post-transcriptional programs support or stabilize reduced mitochondrial activities, thereby facilitating the metabolic readjustment to prolonged low oxygen availabilities [24].

## 3. Discussion

Our study provides a comprehensive picture of total mRNA expression changes (DGE), with contributing transcriptional (DDNS) and mRNA stability (DSR) changes in the course of hypoxia. Interestingly, more pronounced changes in gene expression occurred under chronic as compared to acute hypoxia. Adaptations to acute hypoxia were almost exclusively reflected by enhanced mRNA expression, resulting from induced transcription, whereas total mRNA dynamics during prolonged hypoxic incubations were characterized by reduced expression of numerous targets. While transcriptional changes also contributed to the observed reduction in DGE, certain adaptive traits, most notably mitochondrial functions, instead appeared to be determined mostly by reduced mRNA stability, which was initiated already during acute hypoxic conditions.

Gene expression changes in response to oxygen deprivation have been characterized extensively [25,26,27,28,29], and only recently has a comparative analysis of mRNA synthesis and decay under hypoxia been carried out [19]. Nevertheless, a comprehensive analysis of mRNA expression determining transcriptional and post-transcriptional changes during the course of hypoxia was lacking. Our approach combined the analysis of differential gene expression (DGE) changes with contributions of differential de novo synthesis (DDNS) and stability regulated (DSR) targets during both short- and long-term hypoxia. Our observation that hypoxia induces marked transcriptional changes corroborates the notion that hypoxia-inducible transcription factors are of major importance to coordinate hypoxic responses [30]. The enrichment of similar functions in all DGE and DDNS changes during both acute and chronic hypoxia further underscores that major phenotypic adaptations to hypoxia are determined transcriptionally. In line with this, the transcription factors HIF-1 and 2 have been shown to be essential for an appropriate adaptation to hypoxia [11]. Tiana and colleagues recently showed that the majority of hypoxia-induced changes at mRNA level resulted from altered transcription during short-term hypoxia in HUVEC cells using a similar approach [19]. In contrast, total DSR changes are dominated by reduced target stabilities under both acute and chronic conditions. While global DSR changes appeared to only marginally affect total gene expression programs (i.e., Myc targets), altered metabolic requirements associated with lower oxygen availability (i.e., reduction in oxidative phosphorylation and fatty acid metabolism) were enriched in downregulated DSR. Focusing on functional DSR only, i.e., downregulated DSR that correlated with downregulated DGE, not only supported this observation, but extended it to an mRNA stability-dependent reduction in mitochondria and associated metabolic functions on a broader scale. In addition, these observations add a post-transcriptional component to the previously reported autophagy-mediated reduction in mitochondria and reduced expression of various mitochondrial proteins under prolonged hypoxia [6,7,8,14], which again was supported by our findings of reduced mitochondrial mass and oxygen consumption rate under chronic hypoxia. Functionally, reduced mRNA stability might serve to prevent the futile production of new mitochondrial proteins and mitochondria under conditions where these are actively degraded. On the other hand, the fact that regulation occurs on a post-transcriptional level should allow for a rapid production of mitochondrial proteins once hypoxic conditions are overcome, thereby allowing for an efficient post-hypoxic recovery of mitochondrial energy production. This might be of great relevance in the context of pathophysiological conditions associated with ischemia-reperfusion, to ensure a rapid normalization of the energetic deficits. Since enhanced activities of the respiratory chain are known to elevate levels of reactive oxygen species (ROS), a rapid recovery of mitochondrial functions during reoxygenation might also prove to be detrimental if protective systems against oxidative stress are not upregulated equally as fast [31]. Thus, it will be interesting to see in future studies how fast mRNA stability-evoked repression of mitochondrial functions is alleviated upon reoxygenation and how this impacts mitochondrial energy and ROS production.

As a side note, functional DSR were further enriched in translation-associated mRNAs, suggesting that reduced mRNA stabilities contribute to reduced translation under hypoxia. Interestingly, while the processes of RNA stability and translational regulation have been shown to be tightly connected [32], coupled or parallel regulatory mechanisms were suggested [19]. In contrast, our data suggest that translational processes are subject to mRNA stability regulation under hypoxic conditions. 

We further identified a small group of compensatory DSR, i.e., targets which, while being upregulated on total mRNA level, had reduced mRNA half-lives. The observation that immune responses experience a compensatory mRNA stability reduction under hypoxic conditions corroborates earlier reports that LPS-induced pro-inflammatory mRNAs are destabilized under hypoxia [33]. Importantly, inflammatory conditions commonly bear hypoxic characteristics due to the reduced oxygen availability in the local niches as a result of the infiltration and activation of immune cells [1,2]. Thus, it can be speculated that the importance of compensatory destabilization of immune response-related targets might be even greater during inflammation, where it could contribute to the resolution of inflammation.

Interestingly, the median global mRNA half-life in THP-1 cells under normoxia (3.71 h) appeared to be substantially shorter than in HUVEC cells (8.7 h) [19]. A similar discrepancy was observed in different murine cells, i.e., while the median global mRNA half-life ranged between 5 h [34] and 9 h [35] in NIH3T3 cells, it was only 3.9 h in mESCs [20]. Thus, not surprisingly, similarly to the cell type-specific transcriptional profiles, mRNA stabilities appear to vary between different cellular contexts as well. Despite the overarching differences, the reduced global mRNA half-lives in response to hypoxia observed in our study corroborate the marked reduction found in HUVECs [19]. Furthermore, we observed substantial differences in the half-lives of the different DSR clusters, where mRNAs that showed increased stability under hypoxia shared an extremely short median half-life (2.3 h). In contrast, clusters containing mRNAs with reduced stability had median half-lives of up to 12.3 h (cluster 5). Notably, the total mRNA expression levels appeared to follow a similar distribution, i.e., the cluster with the shortest median half-life had the lowest median expression (cluster 1) and the cluster with the longest half-life had the highest median expression (cluster 5). Strikingly, the functional, mitochondrial DSR targets had an even higher median half-life under normoxia (16.02 h), which was reduced by almost 50% under acute and chronic hypoxia.

Our finding that the experimental validation of selected half-lives by actinomycin D treatment correlated better with the bioinformatically determined half-lives than those determined by 4sU wash-out is in line with previous observations [23]. Yet, others have shown similarly good correlations for the wash-out approach [20]. Considering the great variability in cell-type-, stimulus-, and even mRNA sub-group-specific half-lives, the selection of an experimental approach to determine global and selected mRNA half-lives as well as the bioinformatics tools needs to be made carefully as recently shown in a comparative analysis of various experimental and bioinformatical approaches [36].

In conclusion, while major gene expression changes during the course of hypoxia result from the enhanced transcription of numerous mRNAs, we provide evidence that the reduction in half-lives of specific groups of mRNAs adds to functional traits such as the downregulation of mitochondrial function. Considering the importance of metabolic rewiring during hypoxia and the detrimental effects of reactive oxygen species during hypoxia, but also during reoxygenation, these findings appear of major importance since they might open new opportunities for intervention.

## 4. Materials and Methods

### 4.1. Chemicals

All chemicals were purchased from Thermo Fisher Scientific GmbH (Dreieich, Germany), if not indicated otherwise. Primers were ordered from Biomers (Ulm, Germany).

### 4.2. Cell Culture

THP-1 cells were maintained in RPMI 1640 containing 10% FCS (Capricorn Scientific GmbH, Ebsdorfergrund, Deutschland), 100 U/mL penicillin, and 100 µg/mL streptomycin in an incubator with 5% CO_2_ (normoxia = N). For hypoxic incubation, cells were transferred into a hypoxia workstation (SCI-tive, Baker Ruskinn, Bridgend, South Wales, USA) with 5% CO_2_ adjusted to 1% O_2_ with N_2_ for either 8 h (acute hypoxia = AH) or 72 h (chronic hypoxia = CH). For all experiments performed under hypoxic conditions, freshly prepared hypoxic media and PBS (Sigma-Aldrich Chemie GmbH, Taufkirchen, Germany) were used, and cells were kept under hypoxic conditions until lysis.

### 4.3. Viability Assay

THP-1 cells were labeled with increasing concentrations of 4-thiouridine (4sU, Biosynth Carbosynth, Staad, Switzerland) for 8 h under normoxic conditions. Every 2 h, fresh 4sU was added without exchange of the medium. After 8 h, medium was removed, and the cells were incubated for further 16 h in 4sU-free medium prior to determining viability using the CellTiter-Glo^®^ assay (Promega GmbH, Walldorf, Germany) as described in the manufacturer’s instructions. The IC10 value was determined using nonlinear regression in GraphPad Prism 8.

### 4.4. SLAM-seq

For estimation of transcriptome-wide changes in de novo synthesis and stability of mRNA, thiol-linked alkylation for the metabolic sequencing of RNA (SLAM-seq) was used as previously described [20] with minor modifications.

Briefly, THP-1 cells were seeded in medium at a density of 3.5 × 10^5^/mL and incubated under normoxic or hypoxic conditions. For estimating de novo synthesis, cells were labeled during the last hour of normoxic or hypoxic incubations with 300 µM 4sU. To analyze RNA stability, cells were labeled with 4 pulses of 30 µM 4sU every 2 h during the last 8 h of the incubations without exchanging the medium in between. For wash-out analyses, cells were washed twice in PBS after 8 h labeling (4 × 30 µM 4sU), resuspended in medium containing 3 mM uridine (Sigma-Aldrich Chemie GmbH), and incubated for additional 1, 3, or 6 h under normoxic or hypoxic conditions. At the respective endpoints, cells were lysed in RLT buffer and RNA was isolated using the RNeasy Plus Mini Kit (Qiagen, Hilden, Germany) according to the manufacturer’s instructions with the modification that dithiothreitol (DTT; Carl Roth GmbH & Co. KG, Karlsruhe, Deutschland) was added in all washing steps (0.1 mM) and to the final eluate (1 mM). Five µg extracted total RNA were alkylated using 10 mM iodoacetamide (in 50 mM sodium phosphate buffer, pH 8, 50% DMSO). After 15 min incubation at 50 °C, the reaction was quenched by addition of 20 mM DTT, followed by subsequent ethanol precipitation. RNA integrity was analyzed with an Agilent 2100 Bioanalyzer using the RNA 6000 Nano Kit (Agilent Technologies Germany GmbH & Co. KG, Waldbronn, Germany). 3′UTR libraries were generated from 500 ng alkylated RNA using the QuantSeq 3′ mRNA-Seq Library Prep Kit FWD for Illumina with the UMI Second Strand Synthesis Module for QuantSeq (Lexogen, Vienna, Austria). Library sequencing (single-read; 75 or 150 cycles) was performed on a NextSeq 500 sequencer using a High Output Kit v2 (Illumina, San Diego, CA, USA).

### 4.5. Read Processing, Mapping and Counting

Illumina BaseSpace was used for Bcl2fastq conversion and demultiplexing of pooled libraries. Quality of fastq files was examined using FastQC [37]. Initially, all fastq files were trimmed to 75 nt followed by quality-, adapter- and polyA-trimming with Cutadapt [38]. Subsequently, the unique molecular identifier (UMI) and linker sequences were removed from the reads, and reads were aligned to the human genome (GRCh38) with Ensembl gene annotation (release 80) using STAR (version 2.7.6a) [39] with the following parameters: --alignEndsType EndToEnd --outSAMattributes MD NH --outFilterMultimapNmax 1 --clip5pNbases 12. The resulting bam files were converted to sam files using SAMtools [40], and mutations in the alignments relative to the reference genome were extracted using the Perl script parseAlignment.pl from of the CLIP Tool Kit (CTK, v1.1.3) [41]. The resulting list specified all found mutations, their locations in the genome, as well as the names of the reads in which they were found. The list was filtered for T-to-C mutations using basic Bash commands and kept in bed file format as described in [42]. Based on the filtered list of T-to-C mutations, de-duplicated reads were separated into two bam files holding reads with and without T-to-C mutation, respectively, using SAMtools and basic Bash commands. Separate bam files were generated for total and T-to-C reads, and transcript counts were determined using htseq-count with default parameters [43] and Ensembl gene annotation (release 80).

### 4.6. Differential Gene Expression Analysis of Total (DGE) and T-to-C (DDNS) Data Set

Differential gene expression analysis was performed with DESeq2 in R [44]. Log_2_-transformed fold changes in genes were shrunken using the estimator “ashr”. Benjamini–Hochberg correction was used to determine adjusted *p*-values (padj). Data were visualized using the R packages ggplot2 [45] and ComplexHeatmap [42]. For generation of heatmaps, read counts of significantly changed genes were corrected for library size using DESeq2 size factors and subjected to a row-wise *z*-score normalization.

### 4.7. Determination of mRNA Half-Lives Using GRAND-SLAM

RNA half-lives were estimated based on “globally refined analysis of newly transcribed RNA and decay rates using SLAM-seq” (GRAND-SLAM [23]). Briefly, GRAND-SLAM extrapolates the new-to-total ratio (NTR) and the corresponding posterior distribution based on SLAM-seq data, which allows RNA half-lives to be estimated. Bam files of the 8 h 4sU-labeled samples (for N, AH, and CH) and, in addition, unlabeled control bam files were used for running GRAND-SLAM using the default parameters. The mode of the posterior distribution for the NTR *π* (output from GRAND-SLAM) was used to calculate half-lives *λ* using the formula $\lambda =\frac{-8\;\log\left(2\right)}{\log\left(1-\pi \right)}$. Transcripts with >0 read counts in all samples and half-lives >0 h and <100 h were included in further analyses. Significant global mRNA half-life changes between N, AH, and CH were determined using a Kruskal–Wallis test with subsequent Dunn’s test. Significant single transcript half-life changes between N, AH, and CH were determined using a one-way ANOVA followed by pairwise *t*-tests with Benjamini–Hochberg multiple testing correction. Data were visualized using the R packages ggplot2 [45] and ComplexHeatmap [42]. For generation of heatmaps, half-lives of significantly changed DSR targets were subjected to a row-wise *z*-score normalization.

### 4.8. Gene Set Enrichment Analysis (GSEA)

GSEA was performed using GSEA v4.2.1 [46,47]. Library-size normalized counts (basemean > 0, for all conditions), T-to-C counts (T-to-C basemean >0, for all conditions), or half-lives (half-lives >0 h and <100 h, for all conditions) were used as input and the hallmark gene set “h.all.v7.5.1.symbols.gmt” was as reference gene set. The hallmark genes sets represent specific well-defined biological states or processes. The permutation type was set to “gene_set” and 1000 permutations were performed.

### 4.9. Functional Annotation Clustering

Functional annotation clustering was carried out using the Database for Annotation, Visualization and Integrated Discovery (DAVID) against the gene sets “GOTERM_BP_ALL”, “GOTERM_CC_ALL”, and “REACTOME_PATHWAY” [48,49]. A list of all detected transcripts (basemean > 0, for all conditions) served as background data set.

### 4.10. Transcription Inhibition Using Actinomycin D

THP-1 cells were seeded at a density of 3.5 × 10^5^ cells/mL and incubated under normoxic, acute, or chronic hypoxic conditions, before de novo transcription was blocked by the addition of 2.5 µM actinomycin D (act D; Sigma-Aldrich Chemie GmbH). RNA was isolated using TRIzol (Thermo Fisher Scientific) according to the manufacturer’s instructions, either before (=0 h timepoint) or 1, 2, and 4 h after the administration of act D. RNA concentration was determined by a Nanodrop ND-1000 spectrophotometer (Peqlab Biotechnologie GmbH, Erlangen, Germany). RNA was reverse transcribed using the Maxima First Strand cDNA synthesis kit (Thermo Fisher Scientific) and qPCR analyses were carried out using the PowerUp SYBR Green Master Mix on QuantStudio 3 and 5 PCR Real-Time Systems (all Thermo Fisher Scientific) using primers against MRPL40 (fwd: GAC CAA GAA GCA AAG GAG CGC T; rev: CCT CTC AGT CTC CTC AAA GGT G), CPT1A (fwd: TCG TCA CCT CTT CTG CCT TT; rev: ACA CAC CAT AGC CGT CAT CA), TOMM34 (fwd: CGG CAA TGA GAG TTT CCG C; rev: TCT GAA GAA CCT TGC GCC TG), and GUSB (fwd: CAT TCC TAT GCC ATC GTG TGG G; rev: GGG GGT GAG TGT GTT GTT GAT).

### 4.11. Experimental Validation of mRNA Half-Lives

RNA half-lives were estimated from act D experiments or from SLAM-seq wash-out data by normalizing C_t_ values or library-size normalized T-to-C counts, respectively, to the 0 h control time points. Linear regression in GraphPad Prism 8 was used to determine RNA half-lives.

### 4.12. Statistics

Statistical analyses were carried out using GraphPad Prism v8.2.1 (GraphPad Software, San Diego, CA, USA) or R v4.0.5 [50].

## Data Availability

The sequencing data presented in this study are available in GEO accession number GSE199947.

## Supplementary Materials

The following supporting information can be downloaded at: www.mdpi.com/article/10.3390/ijms23105824/s1.
