# Supplementary material for: Functional RNA Dynamics Are Progressively Governed by RNA Destabilization during the Adaptation to Chronic Hypoxia
Source: Int J Mol Sci. 2022 May 22;23(10):5824. doi: 10.3390/ijms23105824 (PMC9144826; doi:10.3390/ijms23105824)
Supplement: Supplementary file 1 [file ijms-23-05824-s001.zip › Bauer et al - Supplementary Figures.pdf]

SUPPLEMENTARY INFORMATION

Supplementary Figures

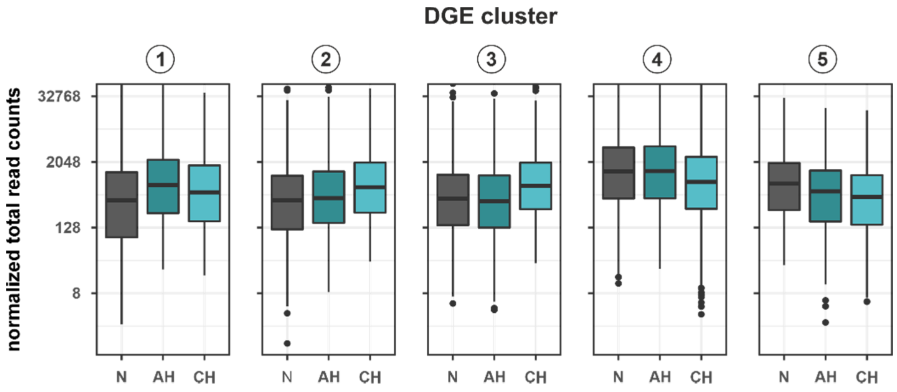

Figure S1: Characterization of DGE clusters. THP-1 cells were incubated under hypoxia (1% oxygen) for 8 h (acute hypoxia, AH) or 72 h (chronic hypoxia, CH), or under normoxia (N), supplemented with 300 µM 4-thiouridine (4sU) for the last hour. Differential gene expression (DGE) changes were determined based on changes in total RNA expression (total read counts) and five groups representing different DGE dynamics during hypoxia were identified by *k*-means clustering analysis (see Figure 1). Normalized total read counts (mean of three replicates) of the targets within each of the five DGE clusters under N, AH, or CH are shown as boxplots.

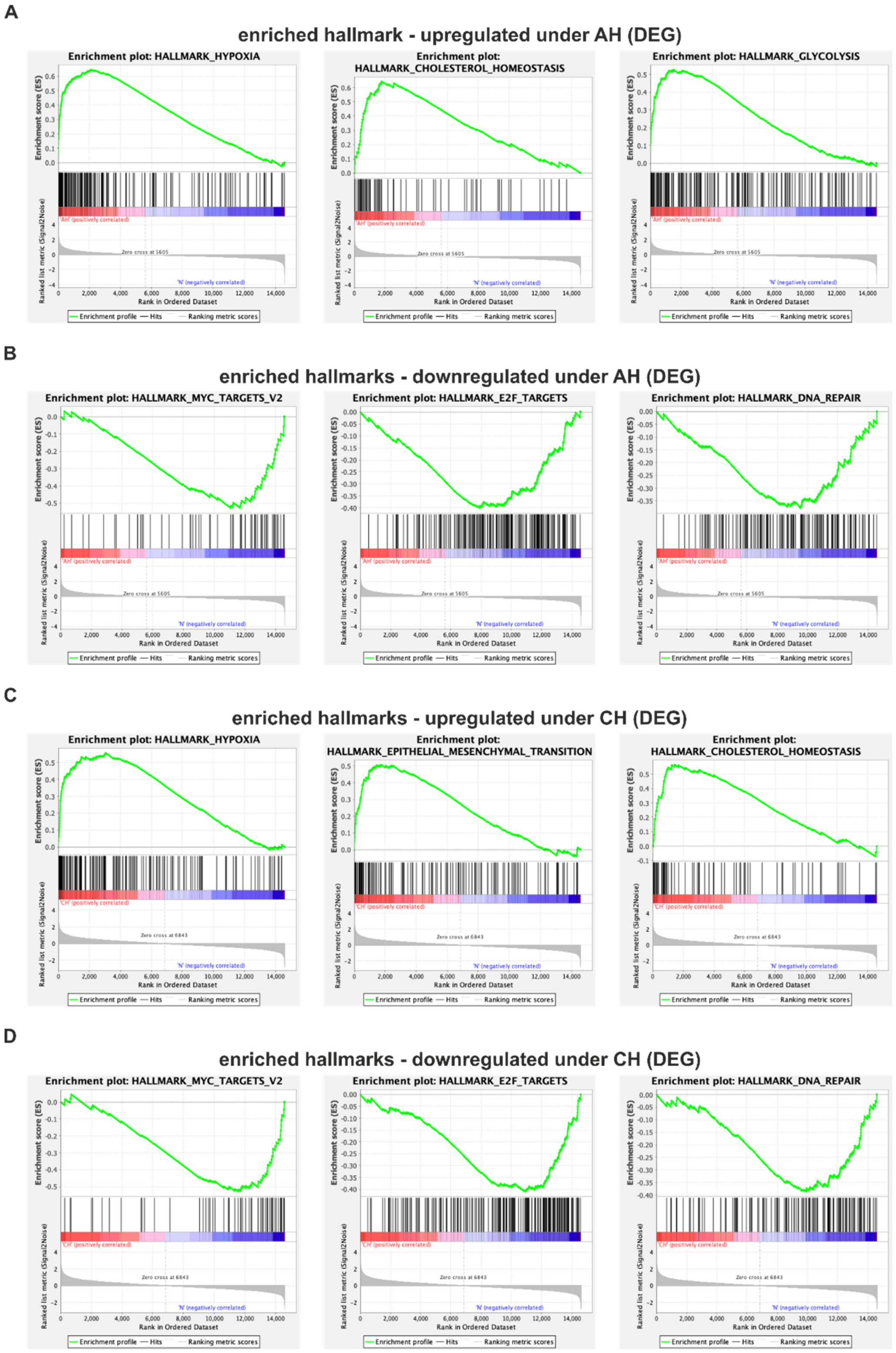

Figure S2: Gene set enrichment analyses (GSEA) of total mRNA expression changes. Top enriched up- (**A, C**) and downregulated (**B, D**) hallmarks as identified by GSEA for DGE regulated between AH and N (**A, B**) or CH and N (**C, D**).

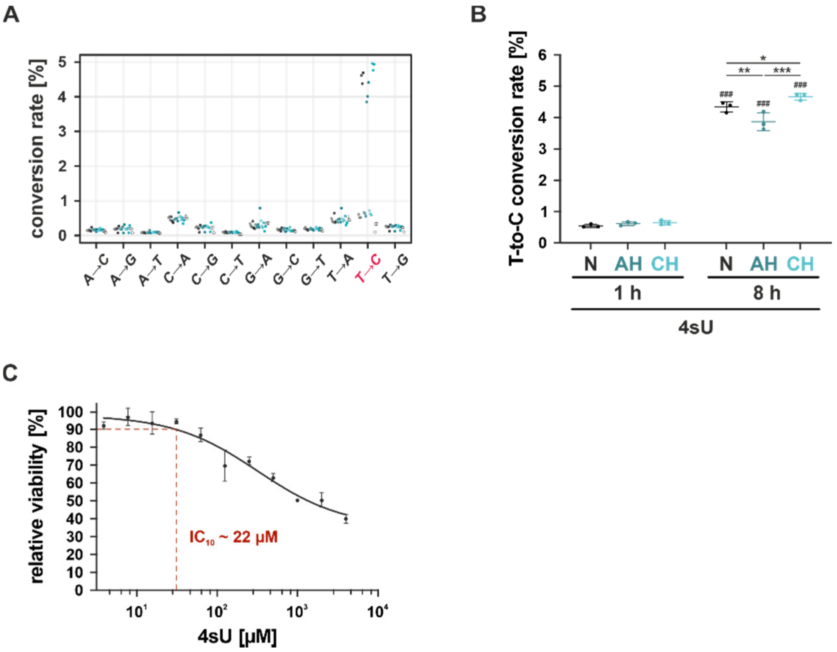

Figure S3: Incorporation and toxicity of 4-thiouridine. (**A, B**) THP-1 cells were incubated under hypoxia (1% oxygen) for 8 h (acute hypoxia, AH) or 72 h (chronic hypoxia, CH), or under normoxia. 4-thiouridine (4sU) was added for either the last hour (300 µM) or the last 8 h (4 x 30 µM 4sU). (**A**) To determine the specificity of the resulting changes, the prevalence of all potential conversions was assessed. (**B**) Incorporation of 4sU in the RNA was further assessed based on T-to-C conversion rates after N, AH, or CH. Residuals were tested for normality using Shapiro-Wilk test, and two-way ANOVA with Sidak's multiple comparison test was performed (* = comparison to N or AH; $^{\#}$ = comparison to 1 h labeling; * p < 0.05, ** p < 0.01, ***/$^{\#\#\#}$ p < 0.001). (**C**) To determine the impact of 4sU on THP-1 viability, cells were labeled with increasing concentrations of 4sU (4 x every 2 hours). Viability was assessed 16 h after labeling using CellTiter Glo® assay and the IC10 was calculated using GraphPad Prism 8.

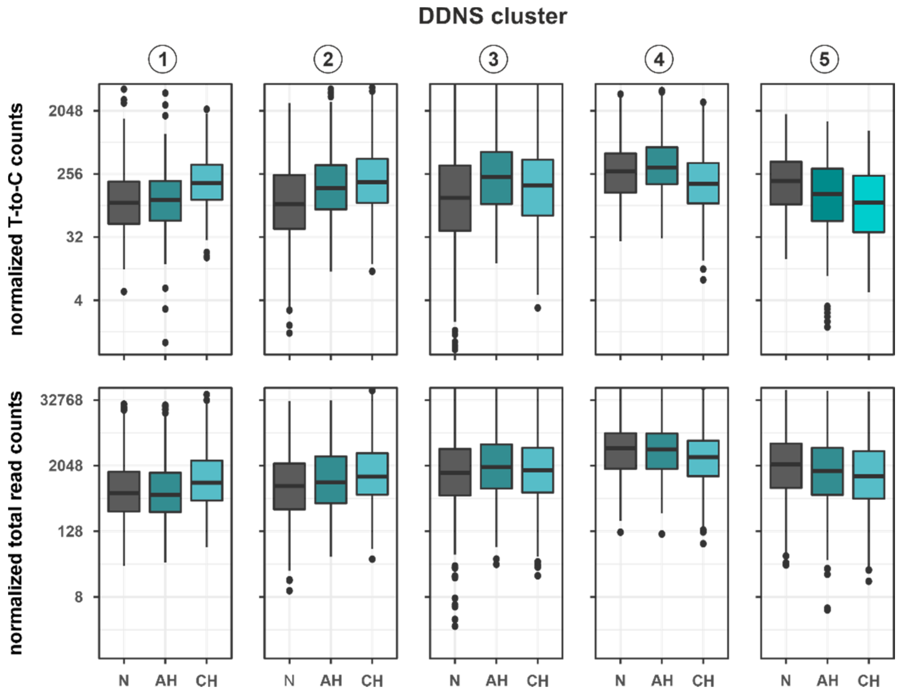

Figure S4: Characterization of DDNS clusters. THP-1 cells were incubated under hypoxia (1% oxygen) for 8 h (acute hypoxia, AH) or 72 h (chronic hypoxia, CH), or under normoxia, supplemented with 300 µM 4-thiouridine (4sU) for the last hour. Differential *de novo* synthesis (DDNS) changes were determined based on changes in T-to-C conversions and five groups representing different DDNS dynamics during hypoxia were identified by *k*-means clustering analysis (see Figure 2). Normalized T-to-C read counts (*upper panels*) and normalized total read counts (*lower panels*) (mean of three replicates) of the target genes within each of the five DDNS clusters under N, AH, or CH are shown as boxplots.

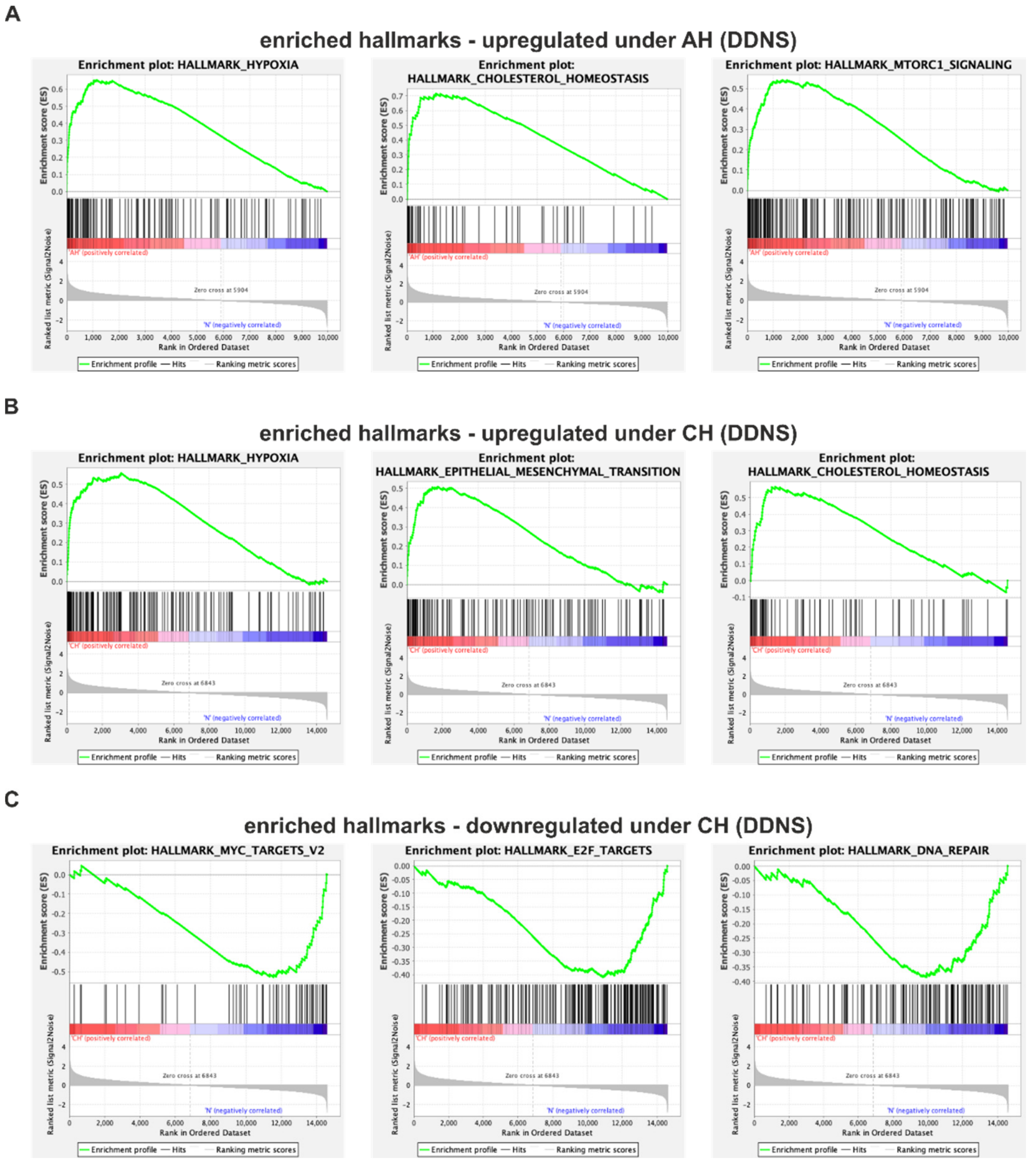

Figure S5: GSEA of T-to-C count changes. Top enriched up- (**A, B**) and downregulated (**C**) hallmarks as identified by GSEA of T-to-C counts comparing acute hypoxia (AH) and normoxia (N) (**A**) or chronic hypoxia (CH) and N (**B, C**).

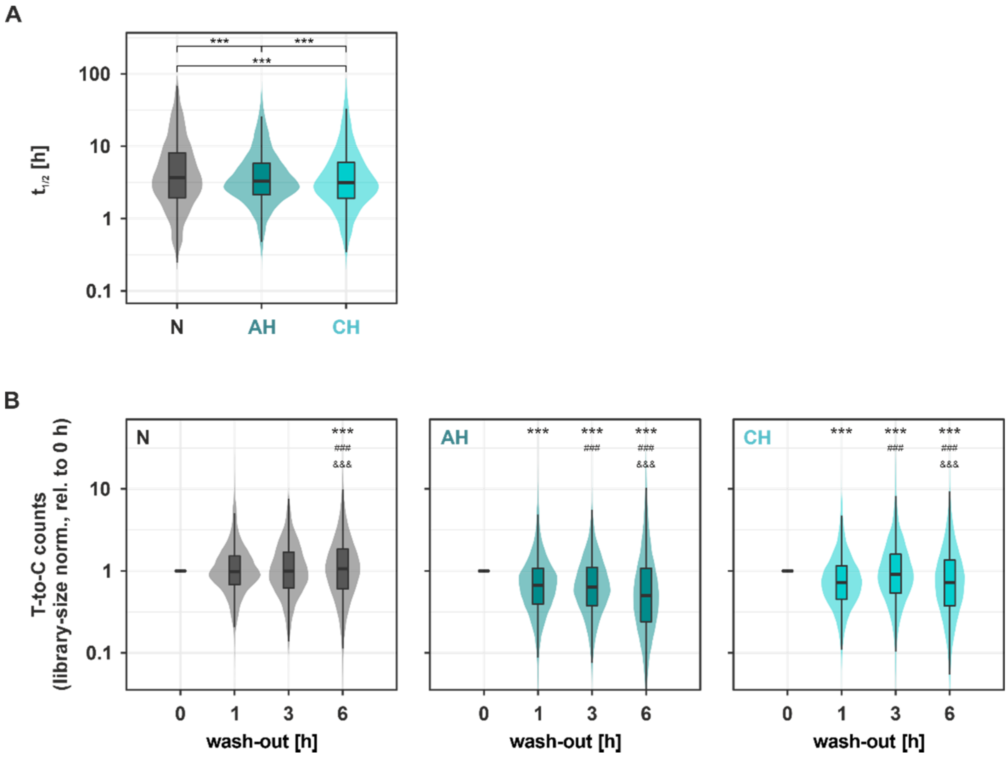

Figure S6: Global stability assessment. THP-1 cells were incubated under hypoxia (1% oxygen) for 8 h (acute hypoxia, AH) or 72 h (chronic hypoxia, CH), or under normoxia, with 4 x 30 µM 4-thiouridine (4sU) during the last 8 h, followed by a washout with excess uridine of up to 6 h. (**A**) Global mRNA half-lives under N, AH, and CH were determined based on T-to-C conversions determined in RNA isolated at the end of the 4sU-labeling using GRAND-SLAM. (**B**) Global washout kinetics under N (*left*), AH (*middle*), and CH (*right*) were determined based on library-size corrected T-to-C counts at the respective washout timepoints normalized to the beginning of the washout. Data were analyzed by Kruskal-Wallis ranked-based test and Dunn's multiple comparison test ($^{***/\#\#\#/\&\&\&}$ $p < 0.001$; compared to 0 h (*), to 1 h ($^{\#}$), and to 3 h ($^{\&}$)).

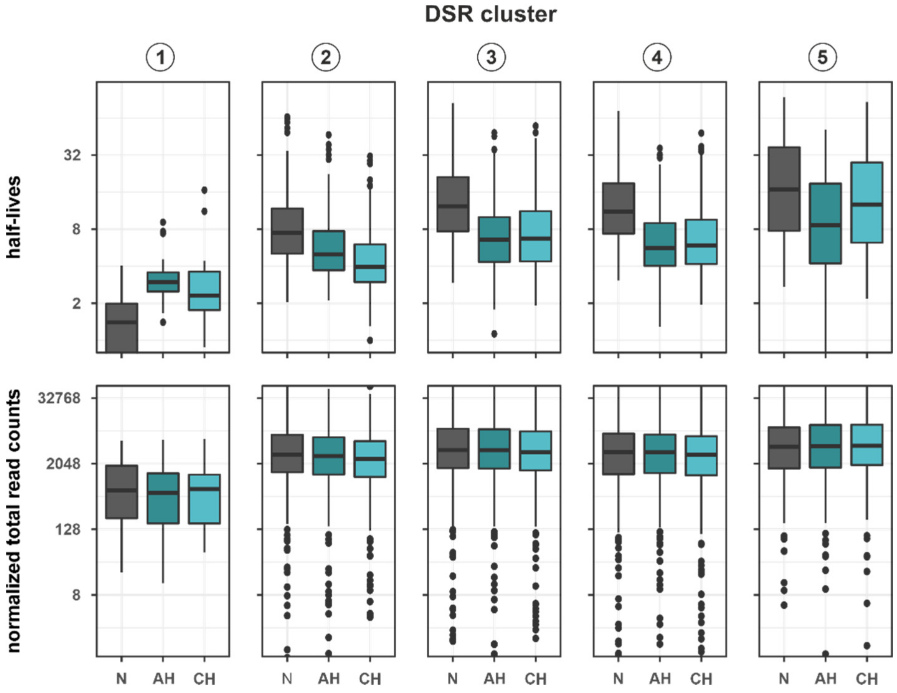

Figure S7: Characterization of DSR clusters. THP-1 cells were incubated under hypoxia (1% oxygen) for 8 h (acute hypoxia, AH) or 72 h (chronic hypoxia, CH), or under normoxia, with 4 x 30 µM 4-thiouridine (4sU) during the last 8 h. Differential stability regulation (DSR) changes were determined based on changes in T-to-C conversions using GRAND-SLAM and five groups representing different DSR dynamics during hypoxia were identified by *k*-means clustering analysis (see Figure 3). Half-lives (*upper panels*) and normalized total read counts (*lower panels*) (mean of three replicates) of the targets within each of the five DSR clusters under N, AH, or CH are shown as boxplots.

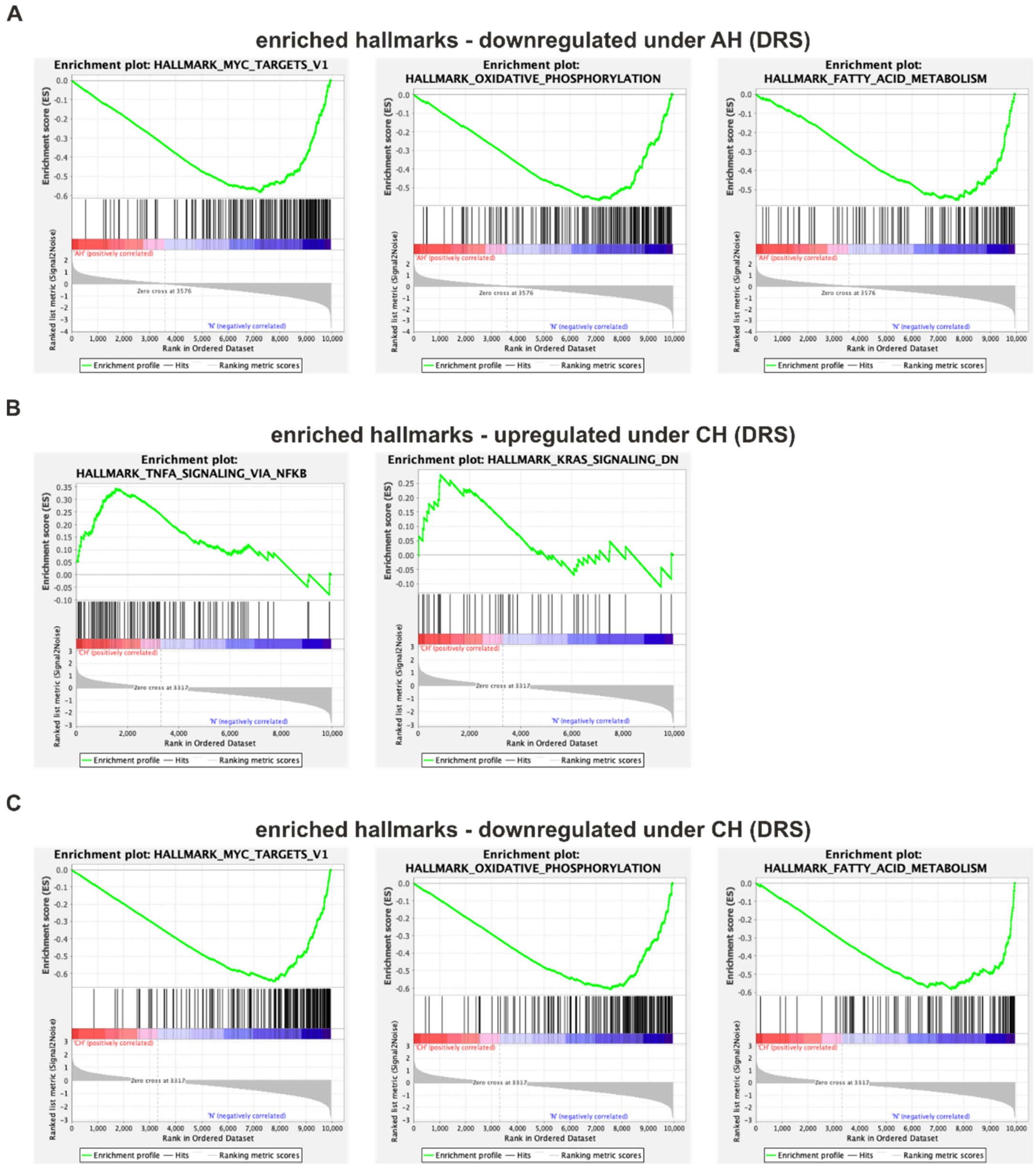

Figure S8: GSEA of mRNA half-life changes. Top enriched down- (**A, C**) or upregulated (**B**) hallmarks as identified by GSEA of half-lives comparing acute hypoxia (AH) and normoxia (N) (**A, B**) or chronic hypoxia (CH) and N (**C, D**).

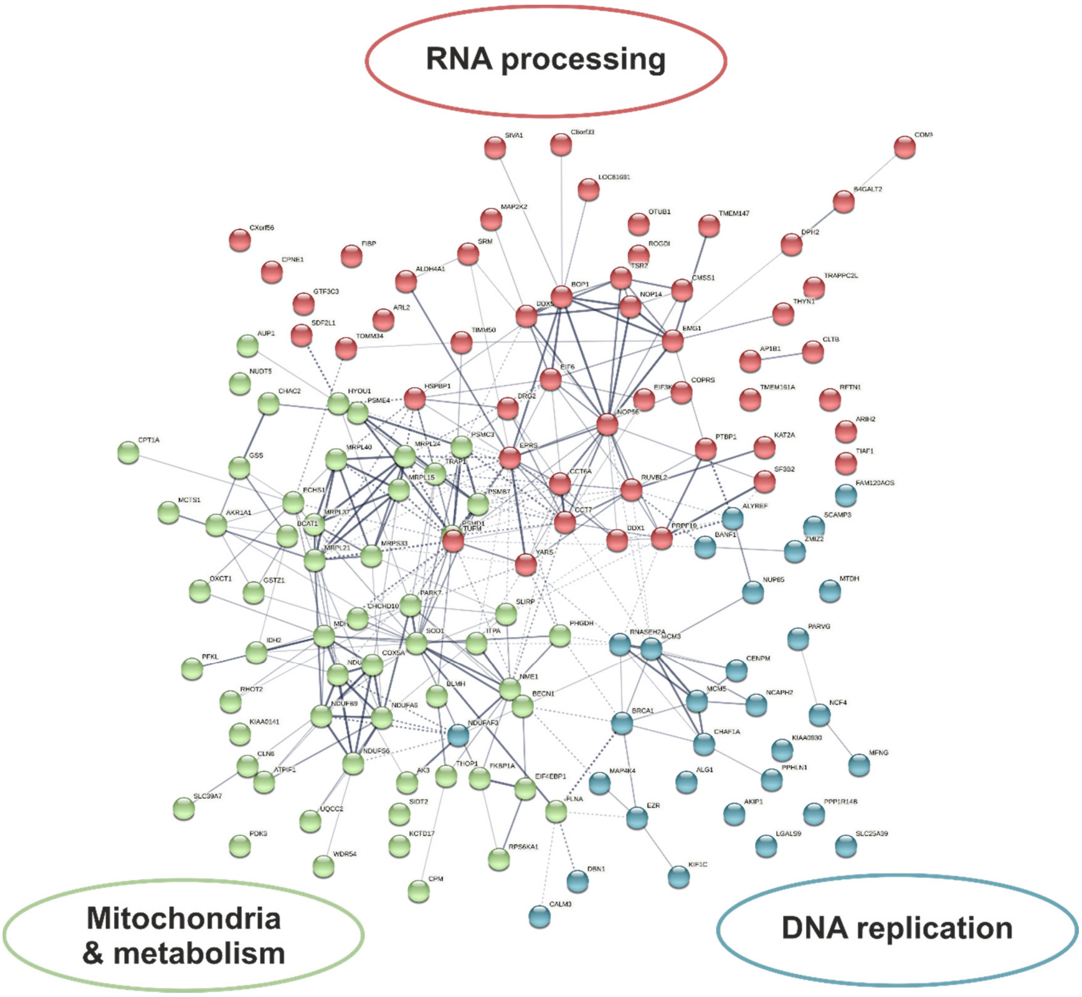

Figure S9: STRING pathway analysis of functional DSR. *K*-means clustering was performed and functional annotations were chosen based on GO terms.

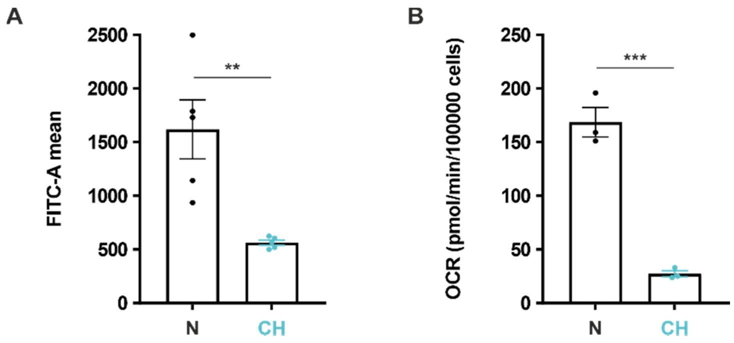

Figure S10: Functional changes in mitochondria under chronic hypoxia. THP-1 cells were incubated under normoxia or chronic hypoxia (1% oxygen, 72 h). (**A**) For relative mitochondrial mass determination, mitochondria were stained with nonyl acridine orange (NAO) prior to FACS analysis. (**B**) The cellular oxygen consumption rate (OCR) was analyzed on a Seahorse extracellular flux analyzer. Data are mean values ± SEM, $n \geq 3$, ** $p < 0.01$, *** $p < 0.001$.

Supplementary Methods

STRING pathway analysis

Biological connections among selected targets were estimated using Search Tool for the Retrieval of Interacting Genes/Proteins (STRING). STRING pathway analysis with *k*-means clustering was performed using default settings (minimum required interaction score: 0.4) [1].

Mitochondrial mass determination

Mitochondrial mass was estimated as previously described [2]. Briefly, after incubating THP-1 cells under normoxia or chronic hypoxia (1% oxygen, 72 h), cells were washed with normoxic or hypoxic PBS and then stained with 20 nM nonyl acridine orange (NAO) diluted in PBS for 30 min in the dark at 4°C to stain mitochondria. Afterwards cells were washed with PBS and analyzed on a LSRFortessa (BD, Heidelberg, Germany).

Cellular oxygen consumption rate determination

The cellular oxygen consumption rate (OCR) was analyzed using a Seahorse 96 extracellular flux analyzer (Agilent, Waldbronn, Germany) as previously described [3]. THP-1 cells were plated on Seahorse 96-well cell culture plates for the measurements, and equilibrated for 30 min before recordings were made in Krebs Henseleit buffer (111 mM NaCl, 4.7 mM KCl, 1.25 mM $CaCl_2$, 2 mM $MgSO_4$, 1.2 mM Na_2HPO_4) supplemented with 5 mM L-glucose and 1 mM L-glutamine.

1. Szklarczyk, D.; Gable, A.L.; Lyon, D.; Junge, A.; Wyder, S.; Huerta-Cepas, J.; Simonovic, M.; Doncheva, N.T.; Morris, J.H.; Bork, P.; Jensen, L.J.; von Mering, C. STRING v11: protein-protein association networks with increased coverage, supporting functional discovery in genome-wide experimental datasets. Nucleic Acids Res. 2019, 47 (D1), D607-D613, doi:10.1093/nar/gky1131.

2. Fuhrmann, D.C.; Wittig, I.; Heide, H.; Dehne, N.; Brüne, B. Chronic Hypoxia Alters Mitochondrial Composition in Human Macrophages. Biochim. Biophys. Acta BBA - Proteins Proteomics 2013, 1834, 2750–2760, doi:10.1016/j.bbapap.2013.09.023.

3. Fuhrmann, D.C.; Olesch, C.; Kurrle, N.; Schnütgen, F.; Zukunft, S.; Fleming, I.; Brüne, B. Chronic Hypoxia Enhances β-Oxidation-Dependent Electron Transport via Electron Transferring Flavoproteins. Cells 2019, 8, 172, doi:10.3390/cells8020172.
